# Barriers and recommendations for colorectal cancer screening in Africa

**DOI:** 10.1080/16549716.2023.2181920

**Published:** 2023-02-23

**Authors:** Rebecca Lee, David Holmes

**Affiliations:** a Department of Family Medicine, Jacobs School of Medicine and Biomedical Sciences, State University of New York at Buffalo, Buffalo, NY, USA; b ECMC Family Health Center, Williamsville, NY, USA

**Keywords:** Colorectal neoplasm, mass screening, colonoscopy, faecal immunochemical test, narrative review

## Abstract

**Background:**

Colorectal cancer (CRC) is the third most common cancer worldwide. The incidence of CRC is rising in low- and middle-income countries but decreasing in high-income countries due to the widespread use of surveillance colonoscopy. In Africa, the implementation of screening programs remains a challenge, even in countries, such as Ghana that have established CRC screening guidelines.

**Objective:**

The purpose of this review was to identify the barriers and recommend strategies for implementing CRC screening in African countries.

**Methods:**

A literature search using PubMed was conducted with the following search terms: colorectal neoplasm, early detection of cancer, mass screening, colonoscopy, faecal occult blood test, faecal immunochemical test (FIT) and Africa. After inclusion and exclusion criteria were applied, a total of 13 articles were reviewed.

**Results:**

The most common barriers reported were limited endoscopic capacity, poor knowledge of CRC and CRC screening, health care factors, cultural factors and sociodemographic factors. Recommendations to increase the availability of CRC screening tests were to include the use of FITs, to provide more training for health care providers, and to expand educational programs for patients, physicians, and religious/community leaders.

**Conclusion:**

The primary barrier to screening for CRC in Africa is the limited endoscopic capacity, specifically the lack of infrastructure and trained personnel, which requires systematic changes by governing bodies. In addition, health care professionals should be involved in educating patients about CRC and CRC screening. Further research is needed to clarify the factors related to subtypes of CRC and to explore the feasibility of using FITs in Africa.

## Introduction

Colorectal cancer (CRC) is the third most diagnosed cancer worldwide [1]. Historically, CRC has been considered a disease of high-income countries and perceived a rare malignancy in low- and middle-income countries such as those in Africa. In high-income countries, such as the United States, CRC incidence rates are decreasing because of the widespread use of surveillance colonoscopy [2,3]. Screening programs detect tumours at earlier stages, which can contribute to a reduction of the incidence and mortality of CRC [4,5]. By contrast, the incidence of CRC is increasing in countries in Africa, including Tunisia [6], Ghana [7] and Nigeria [8]. Moreover, CRC mortality in low- and middle-income countries is attributable to poor awareness of the manifestations of CRC, late diagnosis and the lack of screening tests [9,10]. However, some studies have recommended using scarce resources to combat communicable diseases rather than for CRC screening because of the perceived urgency of the former [11].

Although the World Health Organization does not provide CRC screening guidelines, the World Gastroenterology Organization offers guidelines based on the levels of resources that are available [12]. The Ghana National Cancer Steering Committee recommended the use of the faecal occult blood test (FOBT) as an initial screening tool for patients aged 50–70  years followed by an endoscopic evaluation for those with positive results. However, the impact of this recommendation is not clear because there are limited data on current screening practices [13]. It is also important to note that CRC screening guidelines established in high-income countries may not be applicable everywhere. For example, the age of CRC occurrence in African countries is lower than that in the United States, and there are no universal thresholds for FOBT results for predicting CRC [14–16].

A review of studies reporting on CRC in African countries was conducted to identify the current status of CRC screening and reveal any barriers to and recommendations for implementing CRC screening in Africa. Most of the data were from Ghana and Egypt and thus do not fully represent the state of CRC screening across the continent. However, the findings from these countries were included together in this review due to the lack of data and publications from other African countries. Further research on this topic remains limited and is inadequate to address the rising burden of CRC in Africa.

## Methods

A literature review was conducted using PubMed. The keywords included colorectal cancer screening and Africa. The following MeSH terms were used: colorectal neoplasm, early detection of cancer, mass screening, colonoscopy, FOBT, faecal immunochemical test (FIT), and Africa. Inclusion criteria included articles in English, published between 1990 and 2022, focused on African populations, and reporting on barriers, facilitators or interventions related to CRC screening. Articles that did not meet the stated inclusion criteria or were duplicates were excluded. Article abstracts were initially screened to confirm that the article in review was relevant to the study question and purpose. Information on participants’ demographics, barriers, facilitators and interventions was collected.

## Results

**Figure 1.**
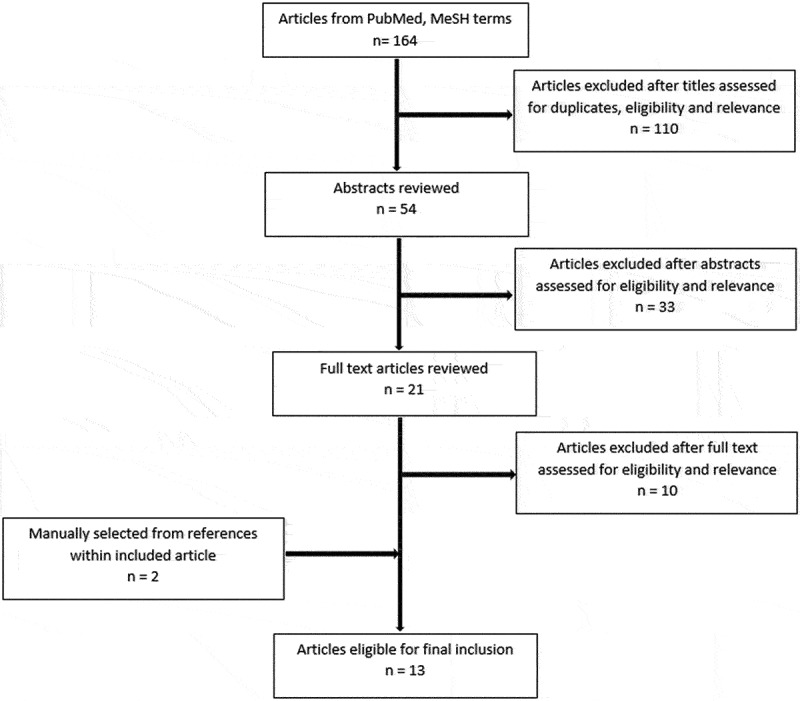
Schematic representation of the article selection process.

The initial search using PubMed yielded 164 articles. Of these, 11 articles met the criteria for inclusion. Two additional articles were selected from citations within these articles (Figure 1). The final review included 13 articles: six were nonintervention studies [13,17–21] (Table 1) and seven were intervention studies [10,22–27] (Table 2).

**Table 1.** Barriers and recommendations for CRC screening in Africa.

Ref. [#], location, and article type/study design	Sample size	Barriers	Recommendations
[20]; Kenya; perspectives paper		Systematic: limited endoscopic capabilities (training, infrastructure), limited no. of trained pathologists (turnaround time, 2–6 weeks)	Train nonphysician endoscopists; use high-resolution micro-endoscopy to improve detection of neoplasia; expand insurance coverage
[18]; Africa; editorial		Systematic: infrastructure, provider availability, cost of screening procedure; Patient: acceptance of screening; Provider: pathologic services, endoscopic training	Consider FIT for CRC screening; consider flexible sigmoidoscopy or serologic blood tests; in-person and virtual training from other specialties and nonphysician providers to increase endoscopic capacity by health care professionals and endoscopic equipment manufacturers
[16]; Egypt; qualitative study, 1-h semi-structured interview	17 physicians (8 specialists, 9 primary care)	Systematic: lack of test availability, cost of screening procedure; Patient: Socio-economic status, fear, cost, cultural belief (only see doctor when symptomatic); Provider: lack of emphasis on prevention, belief that only high-risk patients should be screened, lack of confidence to perform and interpret test, inadequate training for lab technicians and providers	Implement a media campaign emphasising early detection, curability, and prevention; educate physicians and elicit physical engagement; make screening tests widely available; provide well-trained providers; lower cost
[12]; Ghana; qualitative study	39 physicians	Systematic: lack of equipment/facilities, high screening costs/lack of insurance coverage; Patient: lack awareness of early screening benefits, not perceiving CRC as a serious health threat, poor adherence; Provider: not recommending CRC screening for asymptomatic average-risk patients, lack of training/personal adherence to screening	Government to make sustained commitment and financial investment for CRC screening; further studies on true incidence of CRC and individual and societal cost of CRC screening to motivate stake holders to support CRC prevention on a national scale; further studies to understand the exact beliefs and level of knowledge held by the Ghanaian population
[17]; Ghana; qualitative study, interview to understand factors driving screening adherence	14 patients, 14 physicians	Systematic: access to healthcare, lack of national prioritisation; Patient: sociocultural factors, reliance on alternative medicine/religion, perception of stool collection for FOBT, stigma associated with diagnosis of cancer, financial burden, lack of medical education related to CRC and CRC screening; Provider: lack of education/training, not emphasizing importance of screening and fatality of cancer	Leverage mobile health platform for physicians, community, and religious leaders to educate community members; utilise electronic tools such as WhatsApp (90% of information flow)/Qualtrics to train community health works to administer FOBTs at home and to locate nearest endoscopy center; reduce financial burden; systematic change to improve access and national support
[19]; Egypt; intervention mapping, literature review, focus groups, interviews	58 patients, 17 physicians (primary care, gastroenterology, oncology)	Systematic: cost, lack of accessibility, inadequate training among laboratory providers; Patient: CRC knowledge deficit, fear/anxiety regarding testing and cancer diagnosis; Provider: belief that CRC testing should only be performed for high-risk individuals, concerned about the invasiveness of colonoscopy and how patients perceived screening and their fear of a cancer diagnosis, inadequate training among laboratory providers	One-on-one education by health care professionals about the benefits of screening; train health care professionals in basic CRC knowledge and patient engagement and behavior change methods; involve medical students in educating patients on awareness of risk factors, mortality rates, and how to prevent CRC with healthy lifestyle choices; address cost (low cost or free), availability of screening tests, and time spent on the process (convenience)

CRC, colorectal cancer; FIT, faecal immunochemical test; FOBT, faecal occult blood test.

**Table 2.** Interventions for CRC in Africa.

Ref. [#], location and article type/study design	Sample size, patient demographics	Intervention	Result and patient feedback	Barriers	Recommendations
[24]; Nigeria; prospective review	422 (1 male:2.2 female); mean age, 62 yr; most participants had college-level education	FIT (50 ng/ml)	FIT complete, 375 (88.9%); FIT positive, 38 (10.1%); colonoscopy, 28.9% (11/38)	FIT nonadherent: 11.1% (misplaced kit, traveled out of town, lost to follow-up); priority to manage index illness before CRC; discouraged by friends/family; limited capacity/acceptability of colonoscopy; unwilling to undergo invasive procedure when asymptomatic	Make more FIT kits available, link screening option to generic family health programs, educate people on importance of evaluation after positive screening; need additional studies of barriers to uptake of screening colonoscopy
[22]; Egypt; quasi-experimental design	60 (76.7% female); mean age, 68.1 yr (range: 60–83 yr); married, 56.7%; education: university, 60%; secondary, 33%; positive medical history, 96.7%; no tumor, 66.7%	Nurse-led intervention on knowledge and perceptions regarding CRC among older adults	Significant differences (*p* < 0.001) before and after intervention: ‘good’ knowledge of CRC: before (61.7%), after (96.7%), and 4 weeks later (88.3%); good CRC screening practices before (3.3%) and after (63.3%); perceived confidence to perform CRC screening before (39.0%) and after (98.3%)	Lack of interest from older adults in searching for information; seeking medical help when asymptomatic not a common practice nor a welcomed idea in this culture	Design health-related educational leaflets/posters using simple familiar words about CRC prevention and screening procedures and make them available at clubs, at different health care settings, and via mass media and social media; Replicate the study with a larger sample from different geographical areas to achieve more generalised results
[10]; Egypt; quasi-experimental design	140; mean age, 54.3 yr (study group) and 52.1 yr (controls)	Nurse-led intervention on adults’ health beliefs and screening behaviors toward CRC	Significant improvement of total awareness score in study group (9.39 ± 4.31) vs. controls (5.01 ± 2.93); Significant differences (*p* < 0.001) in total mean health belief score pre- and postintervention		Design effective nursing strategies to address barriers of CRC screening and improve CRC knowledge/awareness to achieve greater adherence
[23]; Nigeria; prospective, single-arm study	379; median age, 51 yr; university education, 64.7%; history of prior cancer screening, 45%; previously discussed CRC screening with physician, 6.6%	FIT (50 ng/ml)	FIT complete, 332 (87.6%); FIT positive, 68 (20.5%); colonoscopy, 89.7% (61/68); those who completed FIT found it easy to use (82%), will recommend to family/friends (100%), and will undergo colonoscopy if free (100%)	FIT positive yet nonadherent to colonoscopy: 7 (10.3%) declined or lost to follow-up	Before implementing FIT, need targeted approach to account for low positive-predictive value in average-risk individuals, also need prospective evaluation of FIT-based screening among individuals with known first-degree relative with CRC in Nigeria; develop multi-tiered risk stratification including positive family history, sex, age, cigarette use; obtain more data on natural history of CRC, molecular profile
[25]; Tunisia; quantitative/qualitative review with interviews	5856	FOBT	FOBT complete, 5865 (100%); FOBT positive, 390 (6.7%); colonoscopy, 18.6% (72/390)	Variable adherence from health care professionals; lack of awareness in general population regarding CRC screening; no acceptability of colonoscopy without sedation; problem of affordability; long appointment delays in public sector	Involve the national health insurance fund in CRC screening tests and colonoscopy reimbursement; establish a performance-based payment modality for health care plan
[21]; Nigeria; cross-sectional study	2330 (958 males, 1,372 females) median age, 57 yr; secondary-level education, 68%; even spread across wealth quintiles	FIT (50 ng/ml)	FIT complete, 2109 (90.5%); FIT positive, 432 (20.5%); colonoscopy, 66% (285/432); patients who completed FIT would pay to undergo FIT (out-of-pocket cost $3.65) (73%) or would undergo FIT if free (89%), found the kit easy to use (89%), and will recommend to family/friends (90%)	Level of education: FIT incomplete more in younger (45–54 yr), poorer, less educated participants; higher education level significantly associated with higher rate of colonoscopy attendance; cost: colonoscopy cost equivalent to half of the median monthly household income reported; paucity of endoscopic resources	Higher threshold to account for many false-positive FIT results, design, delivery, and financing of any formal screening program in Nigeria; formal cost-effectiveness analyses and comparative cost-effectiveness analysis are needed for screening programs
[26]; Morocco; demonstration project	9908; age range, 50–75 yr; 10 primary care health centers in 2 providences	FIT (40 ng/ml)	FIT complete, 9763 (98.5%); FIT positive, 460 (4.7%); colonoscopy 62.6% (288/460); patients who completed FIT will recommend the test to family/friends (99%) and found it easy to complete (82%) but messy (52%)	FIT positive but nonadherent to colonoscopy: competing life priorities (15.4%), other health problems (13%), fear of getting cancer diagnosis (12.3%), perception of a low risk of CRC (11.1%); 63% unaware colonoscopy free of charge at research site; increased colonoscopy referral led to increased waiting time and drop in adherence	Media campaign, health education, readiness to get screened for those who feel at risk; train physicians in doctor-patient communication, CRC risk factors, screening, diagnosis, and treatment; promote screening tests in clinics, ensure tests are affordable, provide adequate equipment and training for clinic and laboratory staff, and implement compulsory screening programs; train nonphysician providers to perform the test and to use qualitative FIT (avoids the need for a lab setup)

CRC, colorectal cancer; FIT, faecal immunochemical test; FOBT, faecal occult blood test.

### Endoscopic capacity

One commonly reported barrier to CRC screening in African countries was the limited availability of endoscopic resources [13,17–21] (Table 1). Eight studies have identified the lack of infrastructure as a barrier to CRC screening, more specifically the lack of equipment and facilities [13,17,19,21] (Table 1) and the limited capacity of colonoscopy [22,25–27] (Table 2). Rejaibi et al. [26] mentioned that colonoscopy without sedation was not acceptable to participants, highlighting the lack of supplemental resources needed to improve the patient experience. Alatise et al. [22] reported that high numbers of false-positive results from FITs generated a large endoscopic burden for a country with a paucity of endoscopic resources. Similarly, Selmouni et al. [27] reported that increased colonoscopy referral led to increased waiting times and thus reduced adherence to follow-up CRC screening by those with an initial positive test result.

The limited availability of trained health care professionals, such as physicians and/or laboratory technicians and pathologists was identified as a barrier in all nonintervention studies reviewed (Table 1). Lussiez et al. [13] reported that 60.7% of the physicians that were interviewed identified the shortage of trained providers that could conduct follow-up invasive procedures as a barrier and 58.6% identified a shortage of providers trained for screening modalities other than FOBT as a potential barrier. Physicians also reported the lack of equipment/facilities and training as the top two reasons they did not recommend FOBTs for CRC screening in asymptomatic average-risk patients [13]. In the study by Brand Bateman et al. [17], physicians reported their lack of confidence to perform and interpret CRC screening tests, especially endoscopies, as a barrier to CRC screening.

### Patient education: knowledge of colorectal cancer and its screening

Another barrier to CRC screening in Africa was the lack of patient knowledge [13,17,18,20,26,27]. Lussiez et al. [13] reported that 86.2% of physicians interviewed in Ghana identified patients’ lack of awareness of screening, specifically patients not perceiving CRC as a serious health threat, as a barrier to CRC screening. Furthermore, 65.6% of those physicians noted that primary care physicians do not actively recommend screening in their practices and 10% would not recommend CRC screening for asymptomatic average-risk patients, even if their age was in the range specified in the national guidelines. Three other nonintervention studies listed in Table 1 reported the lack of emphasis and education on preventive screening by health care professionals as a barrier [17,18,20]. Brand Bateman et al. [17] found that only a few participants mentioned doctors as an information source, likely because Egyptians do not regularly visit physicians. Some physicians had concerns regarding how patients perceived screening and a cancer diagnosis [20]. In an intervention study [27], 11.1% of participants reported that they felt they had a low risk of CRC as an explanation for why they did not undergo follow-up colonoscopy after a positive result on the FIT. Interestingly, 63% of the participants in that study were not aware that colonoscopy was free of charge at the study site [27].

### Health care factors

Six studies have reported the high cost of screening procedures, especially colonoscopies, as a barrier to CRC screening [13,17,19,20,22,26]. One-third of the Egyptian physicians interviewed by Brand Bateman et al. [17] identified cost as a barrier. In Ghana, 85.7% of interviewed physicians identified high screening costs/lack of insurance coverage as a barrier [13]. In Nigeria, the cost of a colonoscopy is equivalent to half of the median monthly household income, but 73% of the respondents would pay the cost of $3.65 to undergo FIT [22]. Interestingly, the study by Adonis et al. [28] found that only a small proportion (1.1%) of health-insured people in Ghana received CRC screening in the previous 5 years, whereas enrolment in a comprehensive insurance plan (most expensive plan type) was positively associated with screening for breast and prostate cancer.

### Cultural factors

Some of the significant cultural factors influencing CRC screening in Ghana were patients’ beliefs about health care, religion and traditional medicine and the stigma associated with the diagnosis of cancer [18]. Most (~75%) of interviewed physicians reported the perception of stool collection for FOBT and the invasive nature of colonoscopy as barriers [17,18]. Although patients reported that there is no stigma associated with stool collection, physicians reported that people in traditional settings see faecal matter as an abomination with discomfort [17,18]. Four studies mentioned patient acceptance and fear/anxiety of cancer diagnosis as factors that thwart CRC screening guidelines [17,19,20,27]. In Morocco, 12.3% of participants that had a positive FIT result did not obtain follow-up colonoscopy because they feared a cancer diagnosis [27].

In particular, religion strongly impacts how people relate to health care and medicine, and physician consultations upon initial symptom presentation are not typical [18]. A similar cultural attitude is found in Nigeria [25] and Egypt [23].

### Sociodemographic factors

#### Education

Alatise et al. [22] found that less-educated participants were more likely to fail to complete FITs and that education level was positively associated with the rate of follow-up colonoscopy among patients with a positive FIT result. Interestingly, El Sayad et al. [10] found an association between participants’ educational level and awareness, but not knowledge, about CRC. This finding was consistent with that in the study by Hafez [23], in which one-third of the participants had poor to fair knowledge about CRC despite having a university education.

#### Socio-economic status

Physicians interviewed in Egypt identified socioeconomic status as a predisposing intrapersonal barrier to CRC screening [17]. Specifically, some physicians remarked that patients with more education and of a higher social class were generally more knowledgeable about health and thus visited doctors earlier and more often. Furthermore, patients who had better socioeconomic standing were able to seek private clinics that offer more screening procedures, such as FOBT and colonoscopy, than public clinics, where only the next steps may be suggested for patients with related symptoms such as anaemia.

### Recommendations

In five of the studies, a greater availability of tests was recommended as a way to overcome the barriers to CRC screening [17–21]. Brand Bateman et al. [17] also mentioned the importance of well-trained providers to alleviate the endoscopic burden. Parker et al. [21] and May and Anandasabapathy [19] recommended training nonphysician health care providers to increase the endoscopic capacity, including using virtual sessions from international volunteers. Similarly, Lussiez et al. [18] suggested using electronic tools such as WhatsApp and Qualtrics to train community health workers to administer FOBTs at patients’ homes and to help them locate the nearest endoscopy centre. May and Anandasabapathy [19] also recommended further research to validate the performance, feasibility and cost-effectiveness of FIT as a first line for CRC screening.

To improve CRC knowledge among patients, Nawwar et al. [20] suggested that health care professionals and medical students work with patients one-on-one to tell them the benefits of screening and educate them on risk factors, mortality rates, and how to prevent CRC with healthy lifestyle choices. These could be conducted during regular visits to positively influence patients’ health behaviours. Brand Bateman et al. [17] also recommended that physicians engage with the patients to encourage CRC screening and suggested implementing a media campaign that emphasises early detection, curability, and prevention. Lussiez et al. [13] proposed further investigations of the beliefs and knowledge held by the general population.

Three of the studies reviewed specifically recommended lowering the cost of CRC screening [17,18,20]. In Kenya, an expansion of insurance coverage was recommended to reduce the financial burden [21]. Lussiez et al. [13] emphasised that the Ghanaian government must ensure commitment and financial investment to efforts for CRC screening while acknowledging that further study is needed to determine the true incidence of CRC and the societal cost of CRC screening to motivate stakeholders to support CRC prevention on a national scale.

Recommendations were also made for religious leaders to be involved with the education of the community on health care [18].

### Interventions

Seven articles retrieved in this review involved intervention studies (Table 2). Two of the intervention studies were based on education [10,23], and five discussed organised CRC screening programs [22,24–27]. All the interventions positively influenced participants’ awareness/perception, knowledge of CRC, and CRC screening behaviour.

In Egypt, nurse-led interventions targeting the knowledge and perception of CRC had a positive impact on older adults’ health practices regarding CRC prevention [23]. Along with the statistically improved knowledge and perception of CRC, the interventions increased screening from 3.3% to 63.3% of participants. Nurse-led interventions targeting health beliefs and screening behaviours towards CRC improved total knowledge and awareness scores [10]. There was also an improvement in scores assessing perceived seriousness of CRC and cues to action for prevention of CRC [10].

Organised CRC screening programs have been effective, with most study participants undergoing CRC screening via FIT (90–98%) and all eligible participants undergoing FOBT [22,24–27]. In the study by Labaeka et al. [25], the failure for patients to complete a FIT was ascribed to misplacing the kit, travelling out of town or being lost to follow-up. Three studies mentioned feedback from participants who completed FIT [22,24,27]: 82–89% of participants reported that the test kit was easy to use/complete and 90–100% of participants said they would recommend the FIT to friends and family. Knapp et al. [24] also mentioned that 100% of participants who completed the test kit reported a willingness to undergo colonoscopy if it was free (~90% with a positive FIT result underwent follow-up colonoscopy). The follow-up colonoscopy rates in the other studies were much lower, ranging from 18% to 66% [22,25–27] (Table 2).

## Discussion

CRC screening can detect disease processes early so that patients can be referred to endoscopic centers for diagnosis and treatment [4,5]. In the United States, a high-income country, colonoscopies are a common method for CRC screening [29,30]. Even in rural areas, nearly half of the Americans considered eligible for screening according to the guidelines of the United States Preventative Services Task Force are screened, either by FOBT, flexible sigmoidoscopy, or colonoscopy [31]. By contrast, only 1.1% of the health-insured population in South Africa aged 50 years and over are screened for CRC [28].

The review conducted here highlights the barriers to CRC screening in African countries. The primary barrier is the limited endoscopic capacity, with further impedance from poor knowledge/awareness of CRC and CRC screening and health care, cultural and sociodemographic factors. In several countries in Africa, the health care infrastructure lacks adequate equipment, facilities and trained personnel (i.e. endoscopists and laboratory technicians). This precludes the establishment and implementation of CRC screening guidelines in Ghana [18] and Egypt [20]. In Nigeria, the endoscopy units that are available are used primarily to diagnose and treat symptomatic patients [32,33]. There needs to be an adequate number of trained personnel in these units, because a scarcity of endoscopists can also limit endoscopic capacity in upper-middle-income countries such as Thailand [34]. However, CRC screening can be conducted via the FIT, which is easier to administer than colonoscopy and has higher adherence and detection rates than FOBT [14]. Indeed, most of the participants in the review studies reported that the test kits (FIT or FOBT) were easy to use [22,24,27].

The rates of follow-up colonoscopies for patients with positive results from FIT or FOBT varied among the studies reviewed, but the lowest (18.6%) was attributed to the fact that colonoscopy was offered without the option of sedation at the research site [26]. There are many published studies discussing findings of endoscopies in various hospital centres in Africa, which makes it evident that there are limited but working facilities in urban settings that perform endoscopies where patients can be referred after a positive screening test [32,33]. However, further investigation is needed to determine how accessible these facilities are for the general population and if they have the capacity to conduct follow-up procedures.

The lack of awareness about CRC screening, specifically among both physicians and patients [35], also remains a major barrier [13,20]. The is partly attributable to the lack of public health initiatives to emphasise the importance of screening and the fact that providers are not recommending CRC screening for various reasons, including the lack of equipment/facilities and the lack of appropriate training [13]. In Egypt, respondents to questionnaires regarding CRC knowledge did not score higher than 50% of the maximum [10]. However, nurse-led interventions effectively improved patients’ knowledge scores and readiness to improve health (‘knowing about the problem motivated me to follow screening recommendations’). This exemplifies the strong positive correlation between physician recommendation and CRC screening behaviour [36,37] and demonstrates the influence of health care providers. In-person or online education for patients was a recommended way to improve knowledge of CRC risk factors and emphasise the importance of screening [13,17,20].

The financial challenges associated with the lack of insurance or cost of testing are a common barrier to CRC screening, even in high-income countries such as the United States [38,39]. This is more pronounced in Africa. In 2018, the World Bank estimated that two-thirds of the world’s extreme poor population resides in sub-Saharan Africa [40]. In Ghana, the cost of an FOBT ranges from 30 to 50 Ghana cedi (GH¢) where the daily minimum wage is 11.92 GH¢, equivalent to $2.16 [13]. However, this is not the only barrier to CRC screening, because only a fraction of those in Ghana with insurance were screened for CRC [28]. Notably, a follow-up colonoscopy for a positive result is likely to be cost prohibitive; in Ghana, it was estimated to cost between 300 and 1000 GH¢ [41]. In addition, most countries in Africa do not have well-established health insurance systems to cover the cost of care [42]. However, enrolment in comprehensive insurance plans is positively associated with screening for breast and prostate cancers, demonstrating the effect of one’s financial status on seeking screening [43,44]. The discrepancy in the rate of screening for different cancers may be a reflection of the resources available for endoscopy, which is limited in African countries. Thus, national governments and important stakeholders need to further commit to CRC screening initiatives [13].

A main cultural factor that serves as a barrier to CRC screening in African countries is that individuals typically do not seek medical help unless they have symptoms. The lack of regular doctors’ visits limits the interactions between physicians and patients and eliminates the opportunity to recommend screening. Furthermore, religion has a much larger impact on the medical beliefs of community members [17,18]. Thus, religious leaders are crucial for challenging the deep-seated cultural barrier. For example, fatalism is a cultural barrier to CRC screening in South-Asian immigrants in the United States, as many individuals feel they have no control over whether they get cancer or when they will die [45,46]. A patient’s fear of cancer diagnosis is another common barrier to CRC screening in Africa [19,20,27] and in the United States [38,39].

There were conflicting findings regarding whether participants’ level of education impacts CRC screening. Although a higher level of education was associated with better CRC awareness, it was not linked to better CRC knowledge [10,23]. However, the rates of testing with FIT and follow-up colonoscopy were higher among those with more education in Nigeria [22]. This is consistent with a study on Hungarian participants, which showed that those who were well educated were more likely to be informed about CRC risk factors and symptoms [47]. Although only one of the reviewed studies mentioned socioeconomic status as a potential barrier to CRC screening, it is a relevant factor to consider because socioeconomic status can influence educational opportunities and the cost burden of screening and follow-up procedures.

A limitation of this review is the small number of studies available discussing CRC screening in African countries. There were also no studies from countries in central and southern Africa. Another limitation is the inclusion of an editorial and a perspective article, which introduces risk for bias. Furthermore, most of the studies involved higher-income and higher-educated populations that do not represent the general populations in the countries studied. Lastly, this review did not discuss how differences in the histopathology and epidemiology of CRC in African populations versus those from high-income countries impact the development of specific CRC screening guidelines for the African population. Further studies are needed to determine the true incidence, epidemiology and histopathology of CRC in Africa to ensure that CRC screening guidelines are tailored to the test population.

This review highlights FIT as a valid option for first-line CRC screening that needs to be investigated further. In addition, there needs to be guidelines and strategies in place to ensure that those who have a positive result are referred to endoscopic centres for follow-up diagnosis and CRC treatments, which were not discussed in this review. Finally, studies are needed that identify the beliefs and knowledge held by the populations in each area to inform the development of educational projects. Local communities and media platforms can serve as a steppingstone to empower advocacy movements that encourage governments and important stakeholders to commit and invest in CRC screening and treatment.

The bulk of the data reviewed here were from Ghana and Egypt and therefore are not a complete representation of status of CRC and CRC screening on the African continent. However, this fact highlights the importance of further data collection and research on CRC screening in other African countries. Furthermore, this review was based on existing data favouring current guidelines of CRC screening from the World Gastroenterology Organisation [12]. As noted above, additional factors need to be considered when establishing guidelines, such that those debated as optimal in high-income countries need to be considered with care for low- and middle-income countries [48].

## Conclusion

The barriers to the awareness of and screening for CRC in the African population include the lack of infrastructure and trained personnel, which limit endoscopic capacity, and the limited opportunities for trained personnel to educate patients about the importance of screening and the risks associated with CRC. Greater interactions and awareness will help address cultural barriers, such as the reluctance to seek medical care unless you are ill. Physicians and mid-level providers are called upon to actively recommend CRC screening and recruit religious leaders to educate patients with culturally sensitive programs and materials. Additionally, the cost of CRC screening can be reduced with simpler easy-to-use screening tests and appropriate personnel training as well as investments from governments and important stakeholders. These efforts are essential for successful implementation of CRC screening and the establishment of guidelines in Africa.

## Paper context

The incidence of CRC has been rising in African countries, in contrast to high-income countries where established screening guidelines help to decrease the incidence and mortality of CRC. Common barriers to CRC screening throughout African countries are the lack of infrastructure, trained medical personnel, and educational opportunities for patients. Increased training of providers, education for patients/religious leaders, and simpler screening tests, such as FIT are necessary to successfully implement CRC screening in Africa.
